# Metabolomics with LC-QTOF-MS Permits the Prediction of Disease Stage in Aortic Abdominal Aneurysm Based on Plasma Metabolic Fingerprint

**DOI:** 10.1371/journal.pone.0031982

**Published:** 2012-02-24

**Authors:** Michal Ciborowski, Joanna Teul, Jose Luis Martin-Ventura, Jesús Egido, Coral Barbas

**Affiliations:** 1 CEMBIO (Center for Metabolomics and Bioanalysis), Facultad de Farmacia, Universidad CEU San Pablo, Madrid, Spain; 2 Department of Physical Chemistry, Medical University of Bialystok, Bialystok, Poland; 3 Department of Pharmaceutical Analysis, Medical University of Bialystok, Bialystok, Poland; 4 Vascular Research Laboratory, IIS-Fundación Jiménez Díaz, Madrid, Spain; 5 Autónoma University, Madrid, Spain; Brigham and Women's Hospital, Harvard Medical School, United States of America

## Abstract

Abdominal aortic aneurysm (AAA) is a permanent and localized aortic dilation, defined as aortic diameter ≥3 cm. It is an asymptomatic but potentially fatal condition because progressive enlargement of the abdominal aorta is spontaneously evolving towards rupture.

Biomarkers may help to explain pathological processes of AAA expansion, and allow us to find novel therapeutic strategies or to determine the efficiency of current therapies. Metabolomics seems to be a good approach to find biomarkers of AAA. In this study, plasma samples of patients with large AAA, small AAA, and controls were fingerprinted with LC-QTOF-MS. Statistical analysis was used to compare metabolic fingerprints and select metabolites that showed a significant change. Results presented here reveal that LC-QTOF-MS based fingerprinting of plasma from AAA patients is a very good technique to distinguish small AAA, large AAA, and controls. With the use of validated PLS-DA models it was possible to classify patients according to the disease stage and predict properly the stage of additional AAA patients. Identified metabolites indicate a role for sphingolipids, lysophospholipids, cholesterol metabolites, and acylcarnitines in the development and progression of AAA. Moreover, guanidinosuccinic acid, which mimics nitric oxide in terms of its vasodilatory action, was found as a strong marker of large AAA.

## Introduction

Abdominal aortic aneurysm (AAA) is a permanent and localized aortic dilation, defined as aortic diameter ≥3 cm. Its development is connected with oxidative stress, chronic aortic wall inflammation, elastin fragmentation, apoptosis and loss of extracellular matrix, neovascularization, and depletion of smooth muscle cells (SMC) [1], [2]. It is an asymptomatic but potentially fatal condition because progressive enlargement of the abdominal aorta is spontaneously evolving towards rupture [3].

Based on the population screening studies prevalence rates of AAA vary according to age, gender and geographical location [4]. Although it is hypothesized that the incidence of AAAs has begun to fall in Australia and that the admission rate for aneurysm repair was observed to decline in USA [5], ruptured AAAs are still responsible for at least 15000 deaths in the USA each year [6]. Efforts to limit the mortality rate from ruptured AAA depend on AAA early detection and elective repair. The indication for elective repair, based on large-scale randomized trials, is AAA size above 5.5 cm in diameter [7]. Survival of patients with small AAA (<5.5 cm in diameter) was not improved by elective surgical repair, additionally there is currently no well-defined treatment strategy for them [1]. There were studies evaluating the value of statins [8], β-blockers, antibiotics or antiplatelet therapy in reducing AAA progression but further investigations are necessary in this area [6].

Currently aortic imaging methods such as ultrasound (US), magnetic resonance imaging (MRI) and computed tomography (CT) are valid, suitable, and acceptable methods for AAA diagnosis. However, the benefit of early diagnosis of AAA was limited, because 90% of aneurysms detected by these methods were below the currently accepted threshold for surgical intervention. Moreover, small AAAs do rupture occasionally, therefore, if it could be possible to predict which small AAAs most likely require surgical intervention, intervention could perhaps be offered earlier with less morbidity and mortality [6]. Thus, biomarkers may help to explain pathological processes of AAA expansion, and also allow finding novel therapeutic strategies or determining the efficiency of current therapies. The development of novel high-throughput technologies (e.g. genomics/proteomics/metabolomics) could help in the search of novel biomarkers. Genome wide linkage analysis and candidate gene analysis has been performed in AAA patients [9]. Genetic biomarkers provide insights in disease susceptibility, but the information provided is static. Proteomic studies have been recently applied to AAA tissue and plasma samples [10], [11]. Interestingly, one of the identified biomarkers in one of these studies, peroxiredoxin-1, was associated to AAA presence, size and growth. More importantly, peroxiredoxin-1 quantification provided significantly additive value in predicting AAA growth when combined with AAA size [10]. This data highlights the dynamic and clinical usefulness of proteomic studies in the identification of novel biomarkers of AAA.

Metabolomics combining sensitive analytical techniques with multivariate analysis seems to be a good approach to find biomarkers of AAA. One of the approaches of metabolomics is metabolic fingerprinting, which looks into a total profile, or fingerprint, as a unique pattern characterizing metabolism in a particular case [12]. It was reported previously that this methodology can be applied to the differential analysis of metabolites released by normal and pathological arteries as well as intraluminal thrombus (ILT) into culture medium resulting in the successful identification of compounds related to inflammation and oxidative stress [13]. These compounds could have a high probability of later being found in plasma and be useful diagnostic/prognostic biomarkers.

In the present study, plasma samples of patients with large AAA, small AAA, and controls with high-performance liquid chromatography quadrupole time-of-flight tandem mass spectrometry (LC-QTOF-MS) were fingerprinted. The main adventages of this technique are high sensitivity and good potential for biomarker identification. The broad applicability of LC-QTOF-MS to metabolites of all classes justified the choice for the problem under consideration.

## Materials and Methods

### Ethics statement

Patients and controls were recruited in the Fundación Jimenez Diaz Hospital with the approval of the Ethics Committee at the Fundación Jimenez Diaz Hospital. Written informed consent from all participants involved in the study was obtained.

### Blood Collection and Sample Preparation

Blood was collected from two groups of patients with previously diagnosed abdominal aortic aneurysm: small aneurysm (S) (AAA<5 cm) n = 15 and large aneurysm (A) (AAA>5 cm) n = 15, and from healthy subjects (C) n = 11. Characteristics of study participants are presented in Table 1. For data modeling only 11 samples per group were used in such a way that individuals were matched in age and sex (71±9, 77±4, and 73±7 years for A, S, and C respectively). Additionally plasma samples from 8 AAA patients (4 from A group and 4 from S) who were not matched in age to the other patients in their groups (significantly different according to Mann–Whitney *U* test, p-value = 0.015 in both comparisons) were also analysed and used for prediction. The range of age of those additional patients was 58–59 years for group A and 85–88 years for S. Thus, in total, plasma samples of 41 humans were investigated.

**Table 1:** Characteristics of study participants.

Group	Hypertension	Diabetes	Dyslipidemia	Cardiopathy	COPD	Statins	ILT
Control	10	5	7	3	1	8	0
Small AAA	8	5	6	3	2	4	0
Large AAA	6	1	7	1	2	4	6

COPD – chronic obstructive pulmonary disease.

ILT – intraluminal thrombus.

All subjects were in a fasting state, and blood samples were taken in the morning (between 8 h00 and 11 h00). Fresh EDTA anti-coagulated blood was centrifuged at 1000×*g* for 10 min at 4°C. Plasma was stored in aliquots at −80°C until the day of analysis.

Protein precipitation and metabolite extraction was performed by adding 1 volume of plasma to 3 volumes of cold (−20°C) mixture of methanol and ethanol (1∶1). Samples were then vortex-mixed and stored at −20°C for 5 min. The pellet was removed by centrifuging at 16 000×*g* for 10 min at 4°C, and the supernatant was filtered through a 0.22 μm nylon filter.

Due to the small volume of plasma available in the study, Quality Control (QC) samples (n = 13) were not prepared by pooling an aliquot of them, as in previous studies, but from a pool of human plasma obtained from healthy volunteers that was available in the laboratory. Each QC sample was prepared independently from this pooled plasma following the same procedure as for the rest of samples, and analyzed only once. QCs were injected at the beginning of the run and after every 3–4 real samples to provide a measurement not only of the system's stability and performance [14] but also of the reproducibility of the sample treatment procedure.

### Metabolic Fingerprinting with ESI-QTOF-MS

The HPLC system consisted of a degasser, two binary pumps, and thermostated autosampler, maintained at 4°C (1200 series, Agilent); 10 µL of extracted plasma sample was applied to a reversed-phase column (Discovery HS C_18_ 15 cm×2.1 mm, 3 µm; Supelco) with a guard column (Discovery HS C_18_ 2 cm×2.1 mm, 3 µm; Supelco) thermostated at 40°C. The system was operated in positive ion mode at the flow rate 0.6 mL/min with solvent A - water with 0.1% formic acid, and solvent B - acetonitrile with 0.1% formic acid. The gradient started from 25% B to 95% B in 35 min, and returned to starting conditions in 1 min, keeping the re-equilibration at 25% B for 9 min. Data were collected in positive ESI mode in separate runs on a QTOF (Agilent 6520) operated in full scan mode from 50 to 1000 *m*/*z*. During the analysis two reference masses: 121.0509 *m/z* (C_5_H_4_N_4_) and 922.0098 *m/z* (C_18_H_18_O_6_N_3_P_3_F_24_) were continuously measured to allow constant mass correction, and obtain the accurate mass. The capillary voltage was 3000 V with a scan rate of 1.02 scan per second; the nebulizer gas flow rate was 10.5 L/min. Samples were analyzed in one randomized run.

### Data treatment

The resulting data file was cleaned of back-ground noise and unrelated ions by the Molecular Feature Extraction (MFE) tool in the Mass Hunter Qualitative Analysis Software (Agilent). The MFE then created a list of all possible components as represented by the full TOF mass spectral data. Exact mass databases quoted below were then searched for hits to identify the compounds.

Primary data treatment (filtering and alignment) was performed with Mass Profiler Professional B.02.00 (Agilent) software. Further data treatment was performed by using MS Excel (Microsoft). Data were transformed by applying common logarithm to intensities in order to approximate a normal distribution. The normality of distribution was assessed using the Wilk-Shapiro test. Differences between plasma metabolites in all comparisons (C *vs* A, C *vs* S, and A *vs* S) were evaluated for individual metabolites by using a *t* test. Unequal variance (Welch's *t* test) was assumed. Accurate masses of features representing significant differences were searched against the METLIN, KEGG, LIPIDMAPS and HMDB databases. SIMCA-P+ 12.0 (Umetrics) was used for multivariate statistical calculations and plotting.

To discriminate the samples between the groups and allocate new objects in the model obtained, partial least square discriminant analysis (PLS-DA) method was used. In addition, to avoid the risk of overfitting, models were validated by cross-validation tool [15].

### Compound identification

The identity of compounds that were found to be significant in class separation (Tables 2, 3, and 4) was confirmed by LC-MS/MS by using a QTOF (model 6520, Agilent). Experiments were repeated with identical chromatographic conditions to the primary analysis. Ions were targeted for collision-induced dissociation (CID) fragmentation on the fly based on the previously determined accurate mass and retention time. Comparison of the structure of the proposed compound with the fragments obtained can confirm the identity. Accurate mass data and isotopic distributions for the precursor and product ions can be studied and compared to spectral data of reference compounds, if available, obtained under identical conditions for final confirmation (HMDB, METLIN, LIPIDMAPS). Confirmation with standards was performed by comparison of retention time, isotopic distribution, and fragments of commercially (Sigma) available reagents with those obtain in real samples. All acylcarnitines (Table 3) were confirmed based on characteristic fragments of 60.08 *m/z* identified as [C_3_H_9_N+H]^+^ and 85.03 *m/z* identified as [C_4_H_5_O_2_]^+^. Lysophospholipids (Table 4) were also confirmed with characteristic fragments of 184.07, 104.11, and 86.1 *m/z* for lysophosphatidylcholines (lyso PCs), and of (M+H−141.02 *m/z*) for lysophosphatidylethanolamines (lyso PEs) as previously described [16].

**Table 2:** Identification of metabolites that were significantly differentiating plasma profiles of AAA patients from controls.

Compound	RT (min)	Measured mass (Da)	Mass error (ppm)	Identification	Change [%](p-value)			CV for QCs [%]
					A vs C	S vs C	A vs S	
Guanidinosuccinic acid	0.7	175.056	1.1	176.064, 130.085, 118.125, 70.064, 60.056	+309 (9·10^−10^)	−16 (0.2)	+388 (2·10^−8^)	14
Hippuric acid*	1.3	179.058	−1.1	180.064, 105.033, 77.04, 51.023	−18 (0.04)	−4 (0.8)	−14 (0.4)	30
Cholanoic acid derivative	15.3	356.27	−4.0	357.278, 339.262, 289.209, 275.201, 247.164, 215.176, 161.129, 135.116, 109.098, 95.085, 81.069	−76 (0.03)	+235 (0.2)	−94 (0.0006)	21
C27 bile acid	18.2	430.306	−6.5	431.31, 413.301, 395.295, 367.297, 269.19, 177.123, 149.093, 109.1, 97.063	−68 (0.009)	−6 (0.6)	−66 (0.01)	11
Biliverdin	10.4	582.244	−6.8	583.258, 523.241, 297.122, 229.162	+67 (0.2)	−25 (0.1)	+123 (0.02)	23
Bilirubin*	7.4	584.26	−6.0	585.267, 299.138	−41 (0.2)	+140 (0.2)	−75 (0.01)	16
Oleic acidOR vaccenic acid	31.3	282.255	−3.8	283.263, 265.251, 247.241, 240.233, 191.176, 177.162, 163.144, 149.131, 135.114, 121.1, 97.1, 83.085, 69.07, 57.07	−16 (0.4)	−28 (0.06)	+17 (0.01)	23
Hydroxydecadienediynoic acid	7.5	180.078	−6.4	181.085, 163.075, 153.054, 139.038, 121.028, 71.049	−54 (0.03)	−63 (0.004)	+26 (0.2)	10
N-Methylethanolamine phosphate OR Aminopropan-2-ol O-phosphate	0.7	155.034	−2.9	156.042, 112.051, 98.917	−8 (0.7)	+16 (0.1)	−21 (0.005)	8
Octadecatrienol	31.3	264.243	7.0	265.251, 247.241, 177.161, 163.146, 149.129, 135.117, 121.098, 109.1, 95.086, 81.069, 69.069, 57.069	−8 (0.8)	−59 (0.04)	+122 (0.04)	16
Sphingosine-1-phosphate	15.5	379.246	7.0	380.254, 264.266, 93.068, 82.065	−43 (0.002)	−26 (0.008)	−23 (0.06)	10
Sphinganine-1-phosphate	16.5	381.262	−6.6	382.269, 284.288, 266.282	−25 (0.1)	−36 (0.00001)	+17 (0.7)	10
PC	30.9	855.568	12.0	856.575, 797.5, 761.524, 673.508, 503.286, 444.219, 184.072, 86.096	−25 (0.03)	−30 (0.1)	−8 (0.4)	23

A vs C - (+)/(−) means increased/decreased abundance in large aneurysm group in comparison to controls, S vs C - (+)/(−) means increased/decreased abundance in small aneurysm group in comparison to controls, A vs S - (+)/(−) means increased/decreased abundance in large aneurysm group in comparison to small aneurysm group. Identity of metabolites marked with asterix (*) was confirmed by analyzis of the standard.

**Table 3:** Identification of acylcarnitines that were significantly differentiating plasma profiles of AAA patients from controls.

Compound	RT (min)	Measured mass (Da)	Mass error (ppm)	Change [%](p-value)			CV for QCs [%]
				A vs C	S vs C	A vs S	
Decanoylcarnitine	8.1	315.239	−6.8	−60 (0.0004)	−36 (0.05)	−37 (0.1)	6
Dodecanoylcarnitine	11.8	343.27	−6.9	−51 (0.2)	−44 (0.01)	−13 (0.3)	15
Tetradecenoylcarnitine	13.4	369.286	−6.5	−66 (0.02)	−67 (0.02)	+1 (NS)	6
Palmitoylcarnitine	18.5	399.332	−6.7	−53 (0.002)	−47 (0.007)	−11 (0.2)	7
Linoleylcarnitine	17.1	423.332	−6.3	−55 (0.0007)	−41 (0.01)	−24 (0.5)	7
Vaccenylcarnitine OR oleoylcarnitine	19.1	425.348	−6.4	−60 (0.0006)	−48 (0.009)	−22 (0.2)	13
Stearoylcarnitine	21.7	427.363	−7.5	−71 (0.05)	−22 (NS)	−63 (NS)	46

A vs C - (+)/(−) means increased/decreased abundance in large aneurysm group in comparison to controls, S vs C - (+)/(−) means increased/decreased abundance in small aneurysm group in comparison to controls, A vs S - (+)/(−) means increased/decreased abundance in large aneurysm group in comparison to small aneurysm group.

**Table 4:** Identification of lysophospholipids that were significantly differentiating plasma profiles of AAA patients from controls.

Compound	RT (min)	Measured mass (Da)	Mass error (ppm)	Change [%](p-value)			CV for QCs [%]
				A vs C	S vs C	A vs S	
Lyso PCs	21.4	479.334	7.0	−45 (0.0005)	−40 (0.1)	−9 (0.004)	16
	18.0	481.314	−6.2	−50 (0.0005)	−15 (0.3)	−40 (0.02)	8
	16.4	493.314	−6.4	−52 (0.0005)	−11 (0.5)	−46 (0.002)	11
	19.3	495.332	−1.2	−55 (0.00009)	−20 (0.04)	−44 (0.001)	9
	22.2	507.365	7.0	−55 (0.0002)	+11 (0.9)	−59 (0.002)	13
	21.6	509.344	−5.6	−53 (0.0008)	−21 (0.1)	−35 (0.03)	30
	16.5	517.313	7.0	−73 (0.004)	−36 (0.01)	−26 (0.08)	18
	17.7	519.331	−3.6	−61 (0.00001)	−32 (0.01)	−43 (0.006)	22
	21.0	521.348	−0.1	−61 (0.00006)	−25 (0.07)	−48 (0.003)	19
	23.7	523.361	−4.9	−62 (0.00005)	−29 (0.02)	−46 (0.002)	14
	15.9	541.313	7.0	−49 (0.001)	−36 (0.03)	−20 (0.7)	26
	19.2	545.344	7.0	−42 (0.005)	−20 (0.2)	−27 (0.06)	12
	25.1	549.376	7.0	−57 (0.0001)	−26 (0.1)	−43 (0.007)	18
	18.3	567.33	−4.6	−45 (0.02)	−40 (0.06)	−9 (0.6)	42
Lyso PEs	19.6	453.283	5.0	−50 (0.2)	−26 (0.03)	−33 (0.2)	11
	18.0	477.284	−2.2	−64 (0.0002)	−35 (0.1)	−45 (0.002)	8
	19.8	479.299	−5.8	−14 (0.0005)	−8 (0.5)	−49 (0.01)	9
	23.8	481.314	−6.2	−55 (0.00007)	−34 (0.005)	−32 (0.03)	14
	19.5	503.299	−4.0	−43 (0.007)	−29 (0.3)	−20 (0.6)	8
	17.5	525.282	−5.8	−31 (0.007)	−32 (0.002)	+1 (0.9)	8
	19.0	527.298	−6.0	−53 (0.001)	−52 (0.09)	−4 (0.2)	20

A vs C - (+)/(−) means increased/decreased abundance in large aneurysm group in comparison to controls, S vs C - (+)/(−) means increased/decreased abundance in small aneurysm group in comparison to controls, A vs S - (+)/(−) means increased/decreased abundance in large aneurysm group in comparison to small aneurysm group.

### Validation of the PLS-DA models

Validation of the partial least square discriminant analysis (PLS-DA) models was performed by cross-validation using the leave 1/3 out approach. The data set was divided into three parts and 1/3^rd^ of samples were excluded to build a model with the remaining 2/3^rd^ of samples. Excluded samples were then predicted by this new model and the procedure was repeated until all samples had been predicted at least once. Each time the percentage of correctly classified samples was calculated.

## Results

### Quality control of the methodology

**Figure 1:**
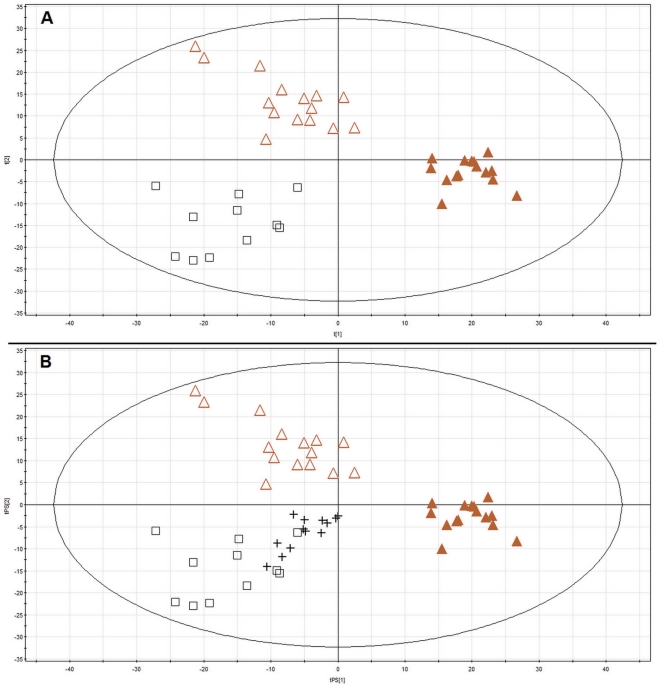
PLS-DA plot of plasma metabolic profiles obtained for patients and controls with prediction for QCs. ▵ - small AAA, ▴ – large AAA, □ – control, + - Quality control Panel A shows PLS-DA model (R^2^ = 0.852, Q^2^ = 0.369) for all samples and all variables in three groups under investigation (A, S, and C). Panel B shows prediction for QC samples by the model obtained.

For quality checking, all 54 chromatograms (41 for patients/controls plasma samples and 13 for QCs) were aligned giving 13050 features in total. A PLS-DA model (Fig. 1, panel A) was built for the three groups under investigation (A, S, and C) taking all variables (without scaling) generated as molecular features in the mass spectrum.

The robustness of the analytical procedure was tested by the prediction of the QC samples by the model (Fig. 1, panel B). Classification of the QCs close to the control group may be explained by the fact, that these samples were prepared from the pooled plasma of healthy volunteers. The quality of the model built for two components was appropriate with variance explained (R^2^ = 0.852), and variance predicted (Q^2^ = 0.369).

### Samples classification

**Figure 2:**
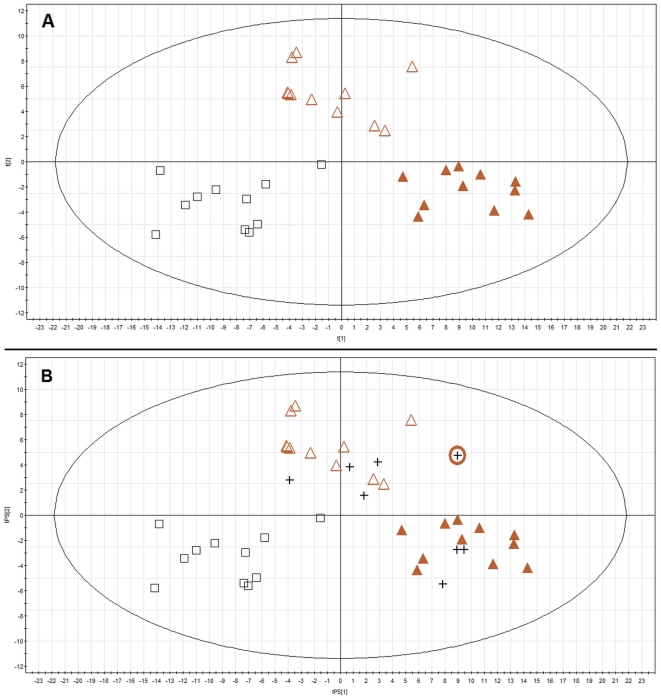
PLS-DA plot of plasma metabolic profiles obtained for age matched aneurysm patients and controls. ▵ - small AAA, ▴ – large AAA, + - predicted AAA samples not matching in age Panel A shows PLS-DA model (R^2^ = 0.835, Q^2^ = 0.335) for plasma samples obtained from patients and controls matching in age (n = 11). Panel B shows prediction for additional samples of patients (4 with large and 4 with small AAA). Sample marked by the circle was obtained from the patient with AAA size of 5.4 cm, and was assigned to large AAA group because the patient was operated on.

To perform sample classification, first all 41 chromatograms were aligned giving primary dataset with 10926 features. However, for further data treatment, only 33 samples of subjects matching in age and sex (n = 11 in each group) were considered. Primary filtering was performed by choosing the data present in a minimum 10 samples in any group, 544 features were selected after filtering. For those 544 features a PLS-DA model with three groups (A, S, and C) was built (Fig. 2, panel A).

The model was validated as described in *Materials and Methods* section. 1/3^rd^ (11 samples) had to be excluded and predicted 8 times until all samples had been predicted at least once. The percentage of samples classified correctly was calculated as 83±6%. In all cases the error was either due to samples from the small aneurysm group misclassified as C or A groups or samples from C or A groups misclassified as S group. As patients with small AAA were at different stages of the disease, some were at an early stage and others at more advanced stage, misclassification connected with this group was strongly possible and does not decrease the validity of the study.

Eight samples from AAA patients that were excluded from the previous model to avoid age mismatch were then predicted by using 544 features selected after primary filtering. As it can be seen on Figure 2, panel B, all samples of patients with small AAA and three samples of patients with large AAA were perfectly classified by the model. Only one sample of a patient with large AAA was classified between A and S groups. Interestingly, after checking the medical records, this patient was found to have the smallest size of AAA among patients in A group (5.4 cm) and was assigned by the medical group to large AAA because the patient was operated on.

To select metabolites that change between groups with statistical significance, first variables underwent secondary filtering to select features present in a minimum of 10 samples *per* group for each comparison separately. Then an unpaired *t* test was performed for log-transformed intensities for comparisons of C *vs* A, C *vs* S, and A *vs* S giving 104, 47, and 61 features with p≤0.05 in each comparison respectively. Identification of statistically significant metabolites was performed as described in *Materials and Methods* section. Identified metabolites are summarized in Tables 2, 3 and 4 including retention time, the mass obtained in the LC-QTOF system and the mass error when comparing with the database. In addition the percentage of change between the groups in the comparisons performed, MS/MS fragments (Table 2), and the coefficients of variation for the abundances of these compounds in QCs (CV) are also shown in the tables.

### Prediction for small AAA

**Figure 3:**
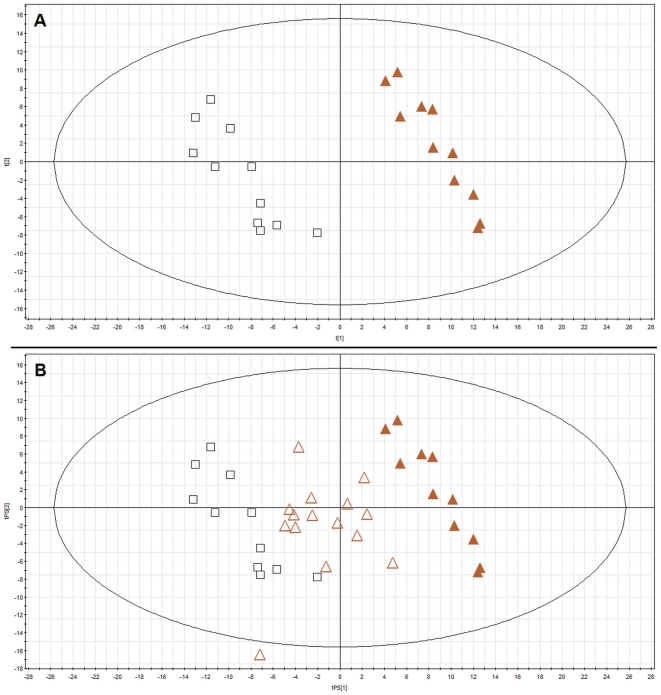
PLS-DA plot of plasma metabolic profiles obtained for age matched large aneurysm patients and controls. ▵ - small AAA, ▴ – large AAA, □ – control Panel A shows PLS-DA model (R^2^ = 0.977, Q^2^ = 0.817) for plasma samples obtained from patients with large AAA and controls matching in age (n = 11). Panel B shows prediction for all samples of small AAA patients.

In addition to the previous model, we wanted to check the ability to predict small aneurysm in a model built for large aneurysms and controls. A PLS-DA model (Fig. 3, panel A) for two groups (A and C) was built for masses after primary filtering (544 variables) and validated as described in *Materials and Methods* section. 1/3^rd^ (7 samples) had to be excluded and predicted 7 times until all samples had been predicted at least once. Samples excluded were always perfectly predicted by the model (100% samples classified correctly).

15 samples of patients with small AAA were then predicted in that model. As it can be seen on Figure 3, panel B, samples of patients with small AAA are classified between controls and large AAA.

These results suggest that there was a clear pattern of metabolites that were detected in the LC-MS plasma fingerprints that need to be investigated.

## Discussion

In the present study some metabolites were detected as significantly altered in plasma, previously found [13] to be released by normal and pathological wall arteries or intraluminal thrombus (ILT) to a culture medium (Table 2). Among them guanidinosuccinic acid (GSA), which was mainly released by the luminal part of ILT, has been found highly increased in the plasma of patients from A group in comparison to controls. GSA, which mimics nitric oxide in terms of its vasodilatory action and its ability to activate the NO generating N-methyl-D-aspartate (NMDA) receptor [17], is probably generated in ILT, secreted to blood stream, and the amount of secreted GSA is related to the stage of AAA. Hippuric acid, which was secreted only by the luminal part of ILT, was found significantly decreased in plasma in group A compared to controls. This observation could correlate to the hyperexcretion of hippuric acid in atherosclerotic state, previously found to be increased in the urine of atherosclerotic rats compared to control group [18]. Other metabolites, like oleamide, stearic acid, docosahexaenoic acid, and alpha-tocopherol were also found to discriminate the plasma of AAA patients from controls, however with significance higher than 0.05 (data not shown).

In the group of other metabolites significantly discriminating the plasma of patients with large aneurysm, small aneurysm, and controls, two (cholanoic acid derivative and bile acid) are metabolites of cholesterol. Decreased levels of those metabolites were observed in plasma of patients from A as well as from S group in comparison to controls. As cholesterol is slightly soluble in water, in plasma it is mainly transported within low-density lipoproteins (LDL) and high-density lipoproteins (HDL). We have also found changes in haemoglobin metabolites (bilirubin, biliverdin). Free haemoglobin could be released to blood stream by haemoaglutination of red blood cells in luminal part of ILT [7].

Long-chain acylcarnitines are decreased in the plasma of aneurysm patients compared to controls. There was a clear decreasing trend with increasing size of aneurysm (Table 3), which may indicate altered fatty acid β-oxidation or deficiency of carnitine. Carnitine is the requisite carrier of activated long-chain fatty acids from cytoplasmic compartment into mitochondria where β-oxidation enzymes are located. There is a significant body of evidence, that carnitine deficiency is associated with many forms of cardiovascular disorders [19], [20], in a study with hypercholesterolaemic rabbits [19] it was shown, that treatment with L-carnitine completely prevented the progression of atherosclerotic lesions. As can be seen in Table 2, a significant decrease in sphingosine 1-phosphate (S1P) and sphinganine-1-phosphate in AAA patients can be found. Both molecules are sphingolipids; sphinganine-1-phosphate is a parent of sphingosine and S1P. Sphingolipids have been suggested to be agonists of peroxisome proliferator-activated receptors (PPARs) [21], [22], which regulate the expression of genes involved in fatty acid β-oxidation and are major regulators of energy homeostasis [23]. The role of PPARα and PPARγ receptors in inflammation and atherosclerosis has been already described, and it is suggested, that PPARs activation regulates various targets that should decrease atherosclerosis, inflammation, and their complications [24]–[26]. Moreover, it was demonstrated, that loss of PPARγ in vascular SMCs promotes AAA in a vascular SMC-selective PPARγ knockout mice model [27]. In addition, S1P was proved to induce vasocontraction in the canine basilar artery *in vitro* and *in vivo*, possibly through a mechanism involving activation of the Rho/Rho-kinase pathway [28]. S1P is a lysophospholipid and significant changes in other lysophospholipids like LysoPEs and LysoPCs are reported in this investigation (Table 4). For those metabolites, similar changes to long-chain acylcarnitines were obserbed, *i.e.* lower concentrations in plasma of patients compared to controls with clear trend to decrease with increasing aneurysm size. The possible explanation for decrease in the amount of LysoPCs/PEs in plasma of AAA patients with a trend related to the size of aneurysm is their accumulation in the ILT. Moreover, in a previous study [13] increased release of LysoPCs by abluminal in comparison to luminal part of ILT was observed. Lysophospholipids are products of the action of phospholipase A2 (PLA2), enzyme hydrolysing phospholipids to free fatty acids, eicosanoids and lysophospholipids. Interestingly, in a very recent report [29] authors showed an increased activity of PLA2 in serum of AAA patients. Eicosanoids and lysophospholipids are very important signaling molecules [30], [31], therefore, increased activity of PLA2 reported by Golledge et al. [29] could be a respond of the body in order to augment low level of lysophospholipids in plasma of AAA patients observed in the present study.

### Conclusion

The results presented here reveal, that LC-QTOF-MS based metabolic fingerprinting of plasma from patients with AAA is a very good technique to distinguish between small AAA, large AAA, and controls which can have a clinical interest. With the use of a validated PLS-DA model it was possible to classify patients according to the stage of the aneurysm and predict properly the stage of AAA of additional patients. Most of metabolites identified in this study were significantly different in the comparison of small AAA with controls, and the tendency to increase the percentage of change and significance with the size of aneurysm. Therefore, identified metabolites could be good targets for the early detection of AAA. These metabolites indicate a role for cholesterol metabolism, carnitine and fatty acid β-oxidation in the development and progression of AAA. Moreover, increased concentrations of sphingolipids observed in the plasma of AAA patients may be responsible for AAA pathogenesis as agonists of the PPAR receptors, and loss of PPARγ in vascular SMCs was found to promote AAA. However, further investigations combining LC-MS with other techniques used in metabolomics like GC-MS or NMR, performed on a bigger population, as well as target analysis of guanidinosuccinic acid with LC-QqQ-MS are necessary to confirm these results and be able to use them as clinical markers.
